# The molecular tumor burden index as a response evaluation criterion in breast cancer

**DOI:** 10.1038/s41392-021-00662-9

**Published:** 2021-07-07

**Authors:** Zongbi Yi, Fei Ma, Guohua Rong, Binliang Liu, Yanfang Guan, Jin Li, Xiaoying Sun, Wenna Wang, Xiuwen Guan, Hongnan Mo, Jiani Wang, Haili Qian, Binghe Xu

**Affiliations:** 1 Department of Medical Oncology, National Cancer Center/National Clinical Research Center for Cancer/Cancer Hospital, Chinese Academy of Medical Sciences and Peking Union Medical College, Beijing, China; 2 Geneplus-Beijing Institute, Beijing, China; 3 Department of Medical Oncology, Huanxing Cancer Hospital, Beijing, China; 4 State Key Laboratory of Molecular Oncology, National Cancer Center/National Clinical Research Center for Cancer/Cancer Hospital, Chinese Academy of Medical Sciences and Peking Union Medical College, Beijing, China

**Keywords:** Breast cancer, Breast cancer

## Abstract

Circulating tumor DNA (ctDNA) is a potential biomarker of prognosis and therapeutic response. We conducted this study to explore the role of the molecular tumor burden index (mTBI) in ctDNA as a therapeutic response and prognostic biomarker in a larger cohort prospective phase III randomized multicenter study. We collected 291 plasma samples from 125 metastatic breast cancer patients from the CAMELLIA study (NCT01917279). Target-capture deep sequencing of 1021 genes was performed to detect somatic variants in ctDNA from the plasma samples. The pretreatment mTBI value was correlated with tumor burden (*P* = 0.025). Patients with high-level pretreatment mTBI had shorter overall survival than patients with low-level pretreatment mTBI, and the median overall survival was 40.9 months and 68.4 months, respectively (*P* = 0.011). Patients with mTBI decrease to less than 0.02% at the first tumor evaluation had longer progression-free survival and overall survival (*P* < 0.001 and *P* = 0.007, respectively). The mTBI has good sensitivity to identify complete response/partial response and progressive disease based on computed tomography scans (88.5% and 87.5%, respectively). The patients classified as molecular responders had longer progression-free survival and overall survival than the nonmolecular responders in the overall cohort (*P* < 0.001 and *P* = 0.036, respectively), as well as in the cohort in which computed tomography scans were defined as representing stable disease (*P* = 0.027 and *P* = 0.015, respectively). The mTBI in ctDNA detected in liquid biopsies is a potential biomarker of therapeutic response and prognosis in patients with metastatic breast cancer.

## Introduction

Despite the great advancements that have been made in solid tumor treatment, tumor metastasis remains the leading cause of cancer-related death. For metastatic cancer, therapeutic monitoring is very important and can help choose the appropriate treatment for patients and avoid ineffective therapies and unnecessary side effects. Serial computed tomography (CT) imaging is generally used to monitor treatment response,^1^ yet imaging examination does not fully represent the pathologic and molecular changes that occur during therapy. The long-term survival of patients with initial radiologically stable disease (SD) is not clear.^1^ A blood biomarker with rapid kinetics could offer an earlier indication of treatment efficacy to help clarify therapeutic management decisions in such cases. Therefore, it is crucial to find biomarkers that assess tumor burden with high specificity and sensitivity.

Repeat tissue biopsy of distant metastases is important for treatment decisions and has been recommended by several recent guidelines,^2–4^ but tissue biopsies are invasive procedures and it is very difficult to detect tumoral heterogeneity due to sample bias.^5^ Circulating tumor DNA (ctDNA) as a method of liquid biopsy could overcome the difficulties associated with tissue biopsy and is representative of tumor heterogeneity.^6,7^ Several studies have shown that ctDNA levels have the potential to be used to monitor treatment response.^8–13^ As a method of quantifying tumor burden, ctDNA has advantages over imaging evaluation. It may distinguish between pseudoprogression and true progression and can be used to evaluate the response of lesions (such as bone metastases) for which radiologic assessment is difficult, and can reduce the dose of radiation applied.^11,12,14^ Moreover, ctDNA can provide molecular information about driver genes, drug resistance genes, and clone structures.^14^ Our group has previously reported that the dynamic changes in ctDNA could reflect changes in tumor burden, and measuring such changes could be used to detect disease progression several weeks earlier than radiographic imaging.^15^

Ongoing challenges to the routine use of ctDNA in clinical practice include clarification of the prognostic and/or predictive associations with anticancer therapy, validation of results in larger patient cohorts, and demonstration of added clinical utility beyond routine radiologic assessment. We conducted this study to explore the role of the molecular tumor burden index (mTBI) in ctDNA as a therapeutic response and prognostic biomarker in a larger cohort prospective phase III randomized multicenter study. We characterized the prognostic and predictive impact of pre-treatment and during-treatment ctDNA analysis. We found that the mTBI in ctDNA can potentially be used as a response evaluation criterion in breast cancer. Molecular response based on the mTBI could predict long-term survival and determine which patients with initial radiologically SD will ultimately respond to anticancer therapy. Furthermore, ctDNA analysis could provide evidence to help make the next treatment choice.

## Results

### Characteristics of patients and sample information

A total of 125 patients with human epidermal growth factor receptor 2 (HER2)-negative metastatic breast cancer treated with first-line chemotherapy were included in this study. The median age of these patients was 46 (ranging from 26 to 72). In addition, 88.0% (110/125) of patients had hormone receptor-positive breast cancer, and 12.0% (15/125) of patients had triple-negative breast cancer. The median number of metastatic sites was 2 (ranging from 1 to 8) and 67.2% (84/125) had visceral metastasis. The other patient characteristics at baseline are summarized in Table 1. ctDNA analyses were performed for each patient at least once. A total of 291 peripheral blood samples were collected from 125 patients. Pretreatment samples were collected from 117 patients and serial plasma samples (with more than 3 samples or including progressive disease (PD) samples) were collected from 30 patients (Supplementary Fig. S1).

**Table 1:** Population characteristics (*N* = 125)

Characteristics	No	Percentage (%)
*HER2 status*		
Positive	0	0.0
Negative	125	100.0
*Hormone receptor status*		
Positive	110	88.0
Negative	15	12.0
*Age at diagnosis*		
≤35	20	16.0
35–60	96	76.8
>60	9	7.2
*Tumor stage at initial diagnosis*		
I	17	13.6
II	41	32.8
III	32	25.6
IV	14	11.2
Unknown	21	16.8
*Nuclear grade*		
1	2	1.6
2	56	44.8
3	20	16.0
Unknown	47	37.6
*Disease-free survival (months)*		
≤12	24	19.2
12–24	17	13.6
24–60	43	34.4
>60	40	32.0
Unknown	1	0.8
*Number of metastatic sites*		
1	33	26.4
2–3	72	57.6
≥4	22	17.6
*Visceral metastases*		
Yes	41	32.8
No	86	68.8
*Previous endocrine therapy (after confirmed tumor relapse)*		
Yes	33	26.4
No	92	73.6

### ctDNA detection in the pretreatment samples

We detected ctDNA in the pretreatment samples of 94 (80.3%) out of 117 patients. The average depth of coverage of the target genes was 1269.9X. Undetectable ctDNA at baseline was associated with a lower disease volume. The mean tumor size (target lesion according to the Response Evaluation Criteria in Solid Tumors (RECIST) version 1.1) in the patients who did not have ctDNA detected at pretreatment was 2.8 cm, which was lower than the 4.5 cm average observed in the patients with detected ctDNA at pretreatment (*P* = 0.045, Supplementary Fig. S2). The median mTBI value at pretreatment in all 117 patients was 2.2 (ranging from 0 to 36.0). The value of mTBI at pretreatment was correlated with tumor burden (*P* = 0.025) but did not correlate with the number of metastasis sites (*P* = 0.060), or visceral metastasis status (*P* = 0.209).

**Fig. 1:**
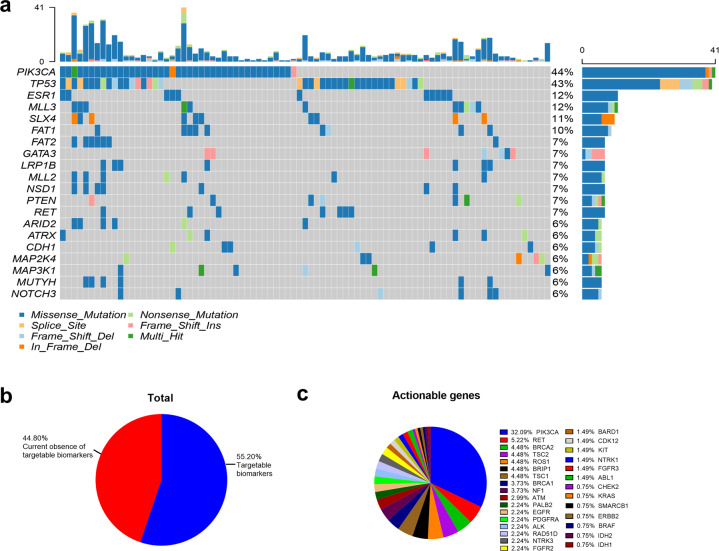
Mutational characteristics. **a** Mutational spectrum of the top 20 genes at pretreatment from 117 patients. **b** Percentage of patients with and without actionable targets for treatment. **c** Genes indicated by the OncoKB knowledge base for which targeted drugs are Food and Drug Administration (FDA)-approved

The commonly mutated genes were *PIK3CA, TP53, MLL3*, and *ESR1*. The frequencies of *PIK3CA, TP53, MLL3*, and *ESR1* mutations were 41 (43.6%), 40 (42.6.2%), 11 (11.7%), and 11 (11.7%), respectively (Fig. 1a).

### Potential clinical implications of somatic mutations in ctDNA

To evaluate whether somatic mutations in ctDNA may be used to improve treatment choices for future patients with metastatic breast cancer, we analyzed which patients could be treated with molecular targeted therapy approved by the Food and Drug Administration (FDA) based on the clinical annotation database OncoKB (https://www.oncokb.org/). A total of one hundred thirty-four mutations were detected, and sixty-nine patients (55.2%) had at least one actionable event for which an FDA-approved drug is currently available; 34.4% (43/125) of the patients had *PIK3CA* mutations in at least one sample and might benefit from alpelisib therapy, which is already approved for breast cancer.^16^ Eleven patients (8.8%) had *BRCA1/2* mutations. These patients might benefit from poly-ADP ribose polymerase inhibitors and/or double-stranded DNA break-inducing chemotherapy. One patient had *ERBB2* mutation and might benefit from anti-HER2 therapies, including neratinib or pyrotinib.^17,18^ In addition, 49 patients (39.2%) had at least 1 alteration predicting response to a drug registered for tumor types other than breast cancer (Fig. 1b-c).

### Prognostic value of pretreatment and early change of mTBI

To assess the predictive value of the mTBI in ctDNA for breast cancer treatment. We further evaluated whether mTBI and clinicalpathological characteristics were associated with survival, including progression-free survival (PFS) and overall survival (OS) rates. First, we analyzed the association of survival and the mTBI value at pretreatment. Bootstrap resampling was used to find an optimized threshold of the mTBI value to stratify by PFS and OS rates.

**Fig. 2:**
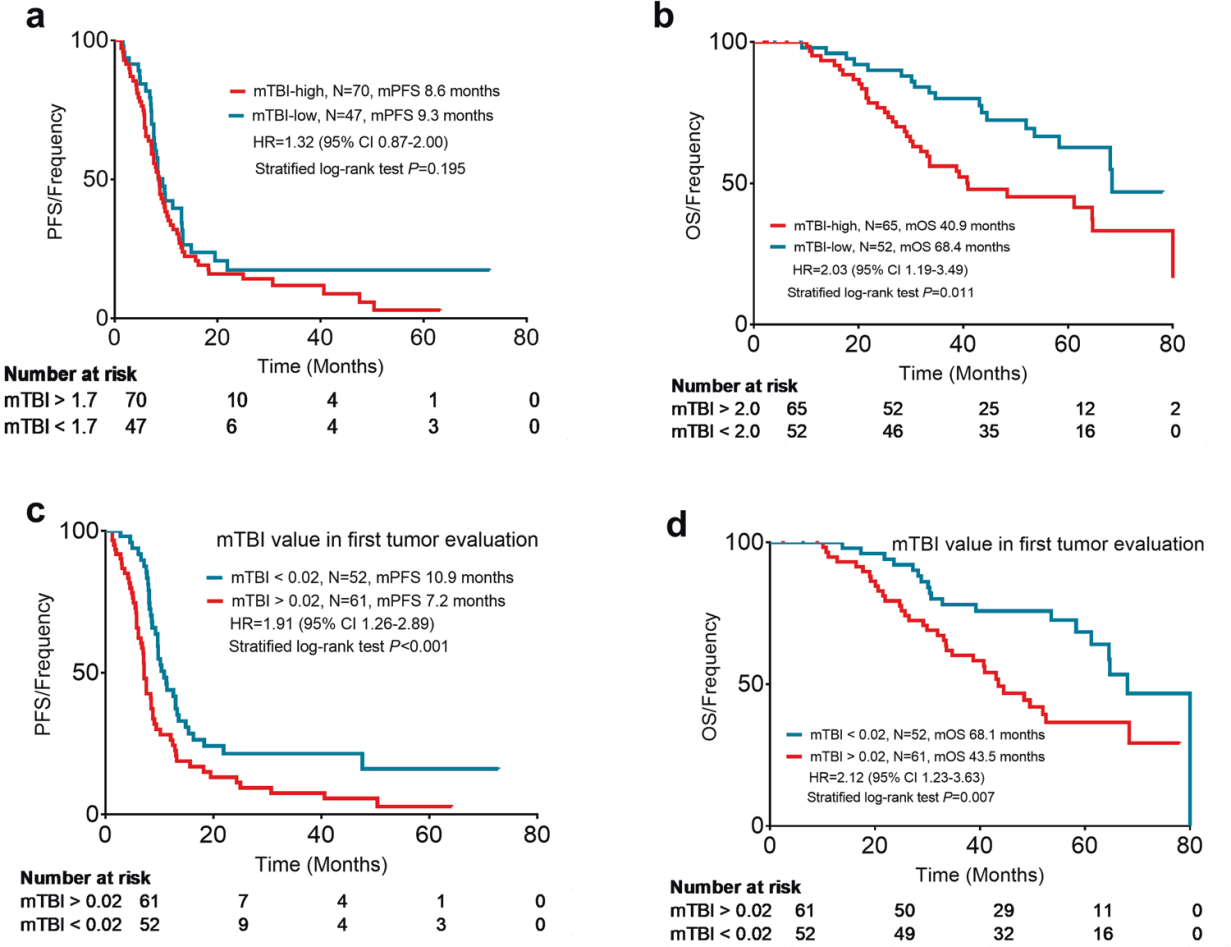
Survival analysis based on the mTBI at pretreatment and first tumor evaluation. **a** PFS analysis based on the mTBI at pretreatment. **b** OS analysis based on the mTBI at pretreatment. **c** PFS analysis based on the mTBI at the first tumor evaluation. **d** OS analysis based on the mTBI at the first tumor evaluation. PFS progression-free survival, OS overall survival, HR hazard ratio, CI confidence interval, mTBI molecular tumor burden index

Patients with a high-level pretreatment mTBI had shorter OS than patients with low-level pretreatment mTBI (mTBI level of 2.0 was the best cutoff), and the median OS was 40.9 months and 68.4 months, respectively (hazard ratio 2.03, 95% CI 1.19–3.49, *P* = 0.011, Fig. 2a). However, the PFS between the mTBI high-level group and mTBI low-level group were not different (mTBI level of 1.7 was the best cutoff), and the median PFS was 8.6 months and 9.3 months, respectively (hazard ratio 1.32, 95% CI 0.87–2.00, *P* = 0.195, Fig. 2b). Then, we analyzed the association between decreasing mTBI levels at the first tumor evaluation and survival. The results showed that patients whose mTBI level had decreased to less than 0.02% at the first tumor evaluation had longer PFS and OS than patients with an mTBI level of more than 0.02% at the first tumor evaluation (median PFS was 10.9 months versus 7.2 months, hazard ratio 1.91, 95% CI 1.26–2.89, *P* < 0.001, Fig. 2c; median OS was 68.1 months versus 43.5 months, hazard ratio 2.21, 95% CI 1.23–3.63, *P* = 0.007, Fig. 2d). In the multivariable analysis, the mTBI value at the first tumor evaluation remained prognostic for PFS and OS rates when controlling for clinical characteristics (see Supplementary Tables S2–3 for full detail regarding univariate and multivariable analysis).

### mTBI evaluated efficacy compared to CT scan

To demonstrate the validity of the measurement, we compared mTBI levels with tumor sizes as assessed by CT. In the receiver operating characteristic (ROC) analysis of mTBI values compared to baseline (pretreatment sample), the area under the curve was 0.98 (*P* = 0.002), suggesting that a decreased mTBI based on serial plasma ctDNA can be used as a predictor of response. From the ROC curve, we defined an mTBI decrease of 80% compared to baseline as a significant change indicating a molecular response.

According to RECIST 1.1, we then grouped the therapeutic responses into four types: molecular complete response (mCR, mTBI reduced to zero during treatment), molecular partial response (mPR, if the baseline mTBI ≥ 1.0%, mPR defined as mTBI decreased by at least 80% compared with baseline; if the baseline mTBI <1.0, mPR defined as mTBI decreased by at least 50% compared with baseline), molecular progressive disease (mPD, mTBI increased by at least 80% compared with the lowest mTBI value, if the lowest mTBI <2, mPD defined as an absolute value of mTBI increased by at least 0.1 compared with the lowest mTBI value), and molecular stable disease (mSD, change in mTBI does not qualify as mPR or mPD).

**Fig. 3:**
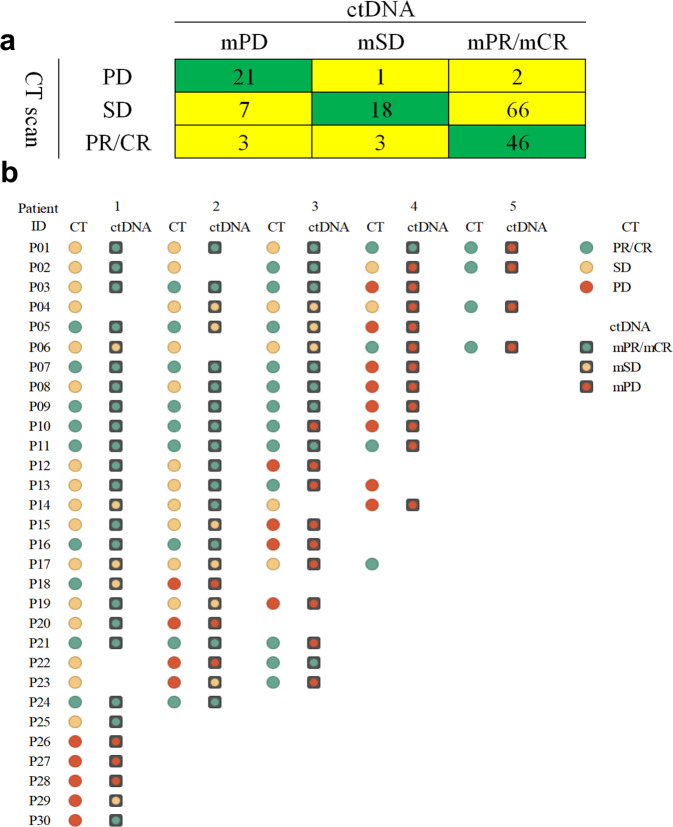
mTBI evaluated efficacy compared to CT scan data. The evaluations based on mTBI values compared with those based on CT scans in all of the patients (**a**) and in 31 patients who had serial plasma samples (**b**). CT computed tomography, ctDNA circulating tumor DNA, mTBI molecular tumor burden index

The evaluations based on mTBI values were consistent with those based on CT scans in 50.9% (85/167) of all the samples (Fig. 3a). Forty-six of the 52 samples (88.5%), which were classified as partial response (PR)/complete response (CR) based on the CT scan, were classified as mPR/mCR based on the ctDNA analysis. Otherwise, 21 of 24 samples (87.5%) classified as PD based on the CT scans were classified as mPD by the ctDNA analysis. This indicates that the mTBI has good sensitivity to identify PR/CR and PD based on the CT scans (88.5% and 87.5%, respectively). However, 72.5% of the 91 samples classified as SD based on CT scans were identified as mPR based on the ctDNA analysis. Moreover, five of the 30 patients (16.7%) who had serial plasma samples had PD detected 6 weeks earlier than the CT scan (Fig. 3b).

**Fig. 4:**
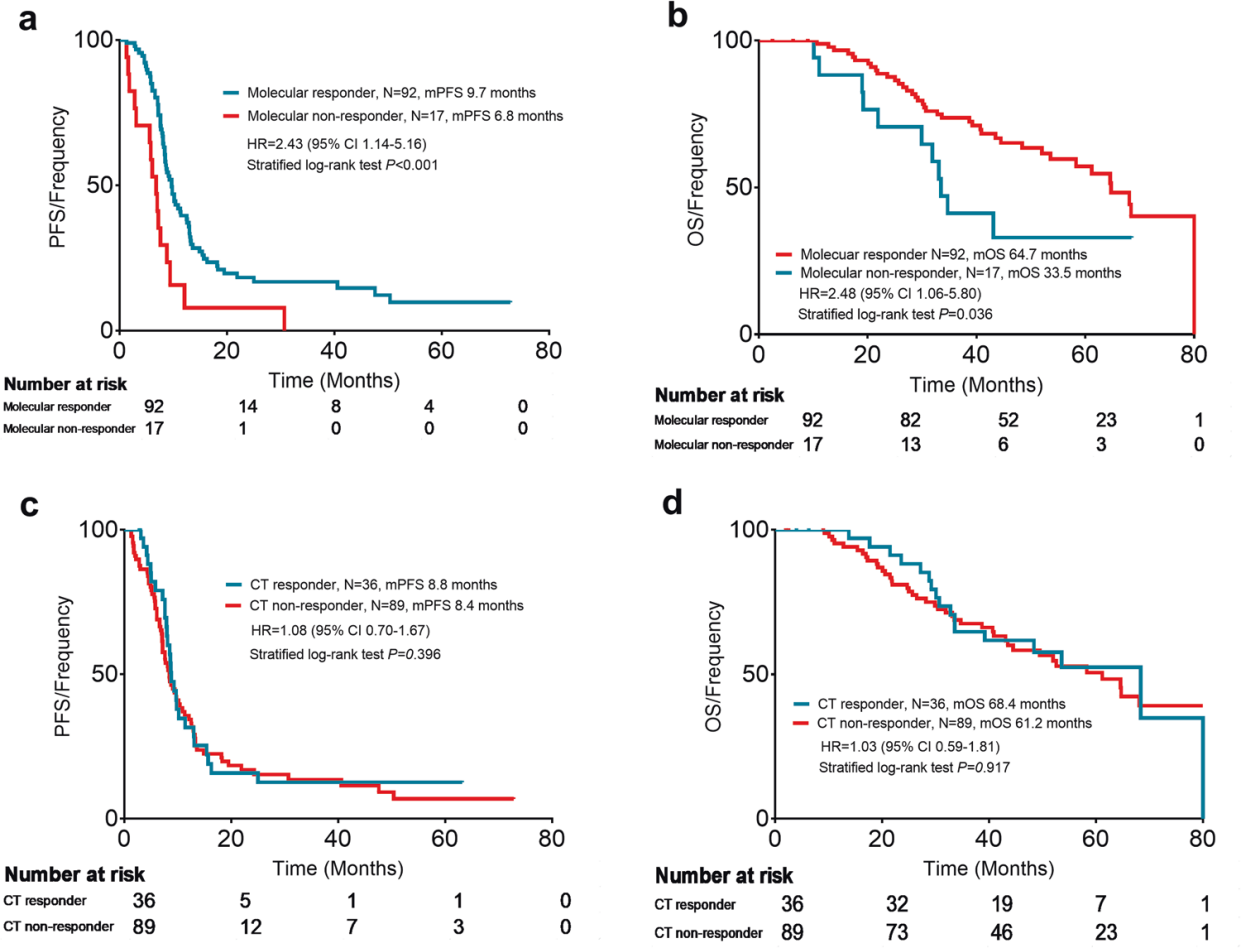
Survival analysis based on early response according to mTBI or CT scan. **a** PFS analysis based on early response according to the mTBI. **b** OS analysis based on early response according to the mTBI. **c** PFS analysis based on early response according to CT scan. **d** OS analysis based on early response according to CT scans. CT computed tomography, mTBI molecular tumor burden index, PFS progression-free survival, OS overall survival, HR hazard ratio, CI confidence interval

**Fig. 5:**
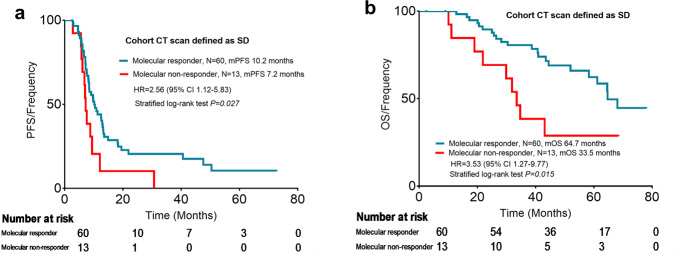
Survival analysis in the cohort CT scan defined stable disease. **a** PFS analysis based on early response according to the mTBI in the cohort with CT scan defined stable disease. **b** OS analysis based on early response according to the mTBI in the cohort with CT scan defined stable disease. CT computed tomography, SD stable disease, mTBI molecular tumor burden index, PFS progression-free survival, OS overall survival, HR hazard ratio, CI confidence interval

Survival analysis was employed to compare the impact on the PFS rate of response in cycle 2 according to the CT scan or our new evaluation criteria. Patients who achieved PR or CR at the first tumor evaluation were defined as responders and those who experienced SD or PD at the first tumor evaluation were defined as the nonresponders. Patients who achieved a molecular response had longer PFS and OS than those of nonmolecular responders (median PFS was 9.7 months versus 6.8 months, hazard ratio 2.43, 95% CI 1.14–5.16, *P* < 0.001, Fig. 4a; median OS was 64.7 months versus 33.5 months, hazard ratio 2.48, 95% CI 1.06–5.80, *P* = 0.036, Fig. 4b). While the PFS between the responders and nonresponders based on the CT evaluation were not different (median PFS was 8.8 months versus 8.4 months, hazard ratio 1.08, 95% CI 0.70–1.67, *P* = 0.396, Fig. 4c; median OS was 68.4 months versus 61.2 months, hazard ratio 1.03, 95% CI 0.59–1.81, *P* = 0.917, Fig. 4d). We further analyzed the survival of the 73 patients who were classified as SD based on CT scans. The results also showed that patients classified as molecular responders had longer PFS and OS than nonmolecular responders in the cohort which CT scans were defined as representing SD (median PFS was 10.2 months versus 7.2 months, hazard ratio 2.56, 95% CI 1.12–5.83, *P* = 0.027, Fig. 5a; median OS was 64.7 months versus 33.5 months, hazard ratio 3.53, 95% CI 1.27–9.77, *P* = 0.015, Fig. 5b).

## Discussion

Response evaluation for anticancer therapy of patients with solid tumors is currently based on radiological assessments.^19,20^ Repeated radiologic assessments have some limitations, such as increased radiation burden for the patient and difficulty assessing bone lesions.^1^ By utilizing highly sensitive next-generation sequencing techniques, ctDNA can be precisely quantified and can provide both opportunities to evaluate tumor response and molecular information.^21,22^ Previous studies indicated that quantification of ctDNA correlates well with tumor burden in solid tumors.^11,23–26^ However, most of the studies focus on some specific genes such as *PIK3CA* and *TP53*, which limits their clinical application. Few studies have been published in which data in comparison with the time of radiological assessment in breast cancer is reported.^27–29^

Our group has previously reported that the mTBI of ctDNA can correlate with changes in tumor burden in response to HER2-targeted therapy.^15^ The mTBI was calculated using the mean allele fraction of mutations in a mutation cluster with the highest cellular prevalence of ctDNA at each time point.^15^ In this study, we fully considered the temporal and spatial heterogeneity of tumors and the characteristics of ctDNA and constructed a new ctDNA-based mTBI algorithm that may be more universally applicable.

Here, we assessed the utility of the mTBI for monitoring tumor response by studying 125 patients from a multicenter study. ctDNA at baseline was detectable in 85% of patients. The low tumor burden in this study may impact the ctDNA detection at baseline. Patients with no ctDNA detected at baseline had a smaller tumor burden. Patients who had no previous chemotherapy were eligible to be enrolled in this study, and 68.8% of patients had no visceral metastases. The results demonstrated that ctDNA analysis has potentially universal applicability for metastatic breast cancer.

In this study, changes in mTBI correlated with target lesion size as evaluated by CT imaging (based on RECIST 1.1). Moreover, we have shown that mutations in ctDNA could be used to improve treatment choices for patients with metastatic breast cancer. We explored the significance of pretreatment mTBI measurements for predicting the PFS and OS rate. We found that the pretreatment mTBI value was associated with OS but not PFS. However, a decreasing mTBI value at the first tumor evaluation was correlated with PFS and OS. Thus, the mTBI value may serve as an early biomarker, reflecting tumor burden changes more quickly than those detected using CT radiography. Decreasing levels of mTBI in ctDNA detected before the appearance of clinical or radiographic tumor shrinkage might aid in the early identification of patients who could achieve a good response to treatment.

Assessment of tumor burden has become an integral part of most oncology clinical trials and can help to evaluate the activity and efficacy of new cancer therapeutics for solid tumors. RECIST 1.1 is widely applied for the assessment of tumor response in therapeutic trials and clinical practice. Outcome events based on RECIST 1.1, such as the PFS rate, can be observed in a short time as a result of accelerating approval of new drugs. However, clinical studies indicated that tumor shrinkage was sometimes not equal to a longer survival time. Patients who achieved a CR or PR based on imaging did not have a good prognosis. Our group and others have previously reported that the levels of ctDNA can correlate with changes in tumor burden in response to anticancer therapy.^10–12,15,23^ This study tested the hypothesis that mTBI values in ctDNA in the plasma of patients with metastatic breast cancer could serve as a potential evaluation criterion of response to anticancer therapy in breast cancer. We established a criterion to evaluate tumor response based on the mTBI of ctDNA compared with CT performance. Accordingly, the response assessed by the mTBI included mCR, mPR, mSD, and mPD. The mTBI has good sensitivity and effectively identifies patients with CR/PR and PD. Patients who achieved mPR based on the ctDNA analysis at the first tumor evaluation had a prolonged treatment response. However, patients who achieved PR based on RECIST 1.1 did not have prolonged PFS and OS. The outcomes of the patients with SD were heterogeneous, some patients have a long PFS, but some patients quickly experienced disease progression. Our results indicated that the ctDNA criterion had the ability to distinguish which of these patients were responders and which were nonresponders.

Currently, no ctDNA test is approved for tumor response evaluation,^14^ and our study indicates that this is a promising field of investigation. ctDNA quantification may be of additional value to distinguish patients who are responders from those who are nonresponders after the radiologically stable disease is reached and to assess bone lesions. Our study revealed that the mTBI of ctDNA could be a potential response evaluation criterion to anticancer therapy in breast cancer. However, RECIST guidelines are also the foundation of response evaluation for solid tumors. Our goal here is to promote conversation related to such issues, not in conflict with the current criteria, but instead, serve as complementary guidance for the most appropriate use of the current RECIST guidelines, and suggest additional details to consider in future clinical trials designs.

In summary, the pretreatment and early change in the mTBI could be a prognostic biomarker for breast cancer patients treated with chemotherapy. The mTBI could potentially be used as a response evaluation criterion and could predict the long-term survival and determine which patients with the initial radiologically stable disease will ultimately respond to anticancer therapy.

## Materials and methods

### Patients and sample collection

The CAMELLIA study (ClinicalTrials.gov Identifier: NCT01917279) was a prospective, randomized, open-label phase III study to explore the efficacy and safety of metronomic chemotherapy with capecitabine versus intermittent capecitabine as maintenance therapy following first-line chemotherapy with capecitabine plus docetaxel in women with HER2-negative metastatic breast cancer at 32 clinical centers in China.^30^ Eligible patients received capecitabine (1000 mg/m^2^ twice daily on days 1–14, every 3 weeks) plus docetaxel (75 mg/m^2^ on day 1, every 3 weeks) for a maximum of 6 cycles or until disease progression, intolerable adverse events, or patient withdrawal occurred. Patients with stable disease or partial or complete response after initial chemotherapy were randomized to receive maintenance chemotherapy with capecitabine of either conventional or metronomic dosage.

The main inclusion criteria were as follows: (1) female patients aged ≥18 years; (2) histologically confirmed and documented HER2-negative metastatic breast cancer; (3) previously untreated first-line chemotherapy (prior hormone therapy for metastatic disease is allowed but must stop before study entry); (4) KPS > 70; and (5) life expectancy of ≥12 weeks. The exclusion criteria were as follows: (1) previous chemotherapy for metastatic breast cancer; (2) prior (radical) radiotherapy for the treatment of metastatic disease or major surgical procedure within 28 days prior to the first study treatment; (3) inadequate bone marrow function; and (4) inadequate liver or renal function. HER2-negative was defined as HER2 membrane staining scored 0 or 1+ by immunohistochemistry or nonamplification by fluorescence in situ hybridization (FISH). ER/PR positive was defined as >1% of tumor cell nuclei staining positively with any intensity by immunohistochemistry.

Serial peripheral blood samples were collected from 125 metastatic breast cancer patients who consented to participate in the biomarker analysis study. Ten milliliters of peripheral blood was collected from each patient before the first treatment, and at every two cycles, at the time of imaging efficacy was evaluated until disease progression. All patients provided written informed consent for this study, and the study was approved by the institutional review board of the Cancer Hospital, Chinese Academy of Medical Sciences.

To evaluate treatment response, CT scans were performed after every two cycles of treatment (three weeks/cycle) or whenever there were signs or symptoms that indicated disease progression according to RECIST 1.1.^19^

### ctDNA analysis

We performed targeted sequencing of 1021 genes that are frequently mutated in breast cancer and other solid tumors. Genes targeted by panels in this study are listed in Supplementary Table S1. Somatic mutations were identified by paired analysis of plasma and germline DNA in blood cells. DNA extraction, library preparation, hybrid capture, sequencing, and analysis were performed as previously described.^18^

### Definition of mTBI

We defined the mTBI based on a comprehensive analysis of somatic variations in ctDNA, considering heterogeneity and dynamic evolution, as follows. We made the extreme assumption that each variant represents subclonal tumor cells. Thus, the number of unique variants detected in a series of plasma samples was considered an important factor. All the exonic nonsilent mutations and InDels detected in a series of plasma ctDNA samples from the same patient were also included in the calculation of the mTBI. Based on these notions, we defined the mTBI as follows:

If a patient had a total of *k* tests, ${\mathrm{mTBI}}_{{k}}({{N}})$ was defined as:

$${\mathrm{mTBI}}_{{k}}({{N}}) = \frac{{\mathop {\sum }\nolimits_{{i}}^{{N}} {\upmu}_{{i}}}}{{{N}}}$$

where ${\upmu}_{{i}}$ is the mutant allele frequency (%) of the position *i*, which was defined as:

$${\upmu}_{{i}} = \frac{{{{B}}_{{i}}}}{{{{A}}_{{i}} + {{B}}_{{i}}}}$$

where ${{B}}_{{i}}$ is the number of mutant reads at position *i* and *A*_*i*_ is the number of wild-type reads at position *i*. Then, all the reads were calculated after duplicates were removed. *N* is the number of unique variants in the mutant point set *S*:

$${{S}} = \mathop {\bigcup}\limits_{{k}} {{{s}}_{{k}}}$$

where ${{s}}_{{k}}$ is the total number of somatic mutants detected at *k* sampling times? *S* is dynamic and is determined by the selected testing period; thus, the mTBI is a relative value. *N* also reflects the heterogeneity of the tumor subclone and ${\upmu}_{{i}}$ reflects the abundance of specific tumor subclone cells.

### Statistical analysis

The PFS was defined as the duration from the date of enrollment to the date of disease progression or death from any cause. OS was defined as the time from the date of enrollment to death from any cause. Patients without an endpoint (progression or death events) were censored at the date of the last follow-up. The last follow-up occurred on May 8, 2020. Kaplan–Meier survival plots were generated based on ctDNA, and curves were compared using log-rank tests. Univariate and multivariate Cox proportional hazards analyses were performed to compare clinical characteristics and ctDNA. Linear regression was calculated to assess the relationship between the mTBI and target lesion size as evaluated by CT imaging and other clinical characteristics. ROC analysis was performed to evaluate the ability of the mTBI to predict treatment response. The cutoff values were evaluated based on the association between patient survival and the mTBI at pretreatment using the survminer R package. All statistical analyses were performed with R v3.6.0, SPSS (v.21.0; STATA, College Station, TX), or GraphPad Prism (v. 6.0; GraphPad Software, La Jolla, CA) software. Statistical significance was defined as a two-sided *P*-value of <0.05.

## Supplementary information

Supplementary Materials

## Data Availability

All data that support the findings of this study are available from the corresponding author upon reasonable request.
